# Maternal Serum Meteorin Levels and the Risk of Preeclampsia

**DOI:** 10.1371/journal.pone.0131013

**Published:** 2015-06-29

**Authors:** María F. Garcés, Elizabeth Sanchez, Luisa F. Cardona, Elkin L. Simanca, Iván González, Luis G. Leal, José A. Mora, Andrés Bedoya, Juan P. Alzate, Ángel Y. Sánchez, Javier H. Eslava-Schmalbach, Roberto Franco-Vega, Mario O. Parra, Ariel I. Ruíz—Parra, Carlos Diéguez, Rubén Nogueiras, Jorge E. Caminos

**Affiliations:** 1 Department of Physiology, School of Medicine, Universidad Nacional de Colombia, Bogotá, Colombia; 2 Department of Internal Medicine, School of Medicine, Universidad Nacional de Colombia, Bogotá, Colombia; 3 Institute of Clinical Investigations, School of Medicine, Universidad Nacional de Colombia, Bogotá, Colombia; 4 Department of Pathology School of Medicine, Universidad Nacional de Colombia, Bogotá, Colombia; 5 Department of Obstetrics and Gynecology, School of Medicine, Universidad Nacional de Colombia, Bogotá, Colombia; 6 Department of Physiology (CIMUS), School of Medicine-Instituto de Investigaciones Sanitarias (IDIS), University of Santiago de Compostela, Santiago de Compostela, Spain; 7 Biomedical Research Centre in Physiopathology of Obesity and Nutrition (CIBERobn), Instituto de Salud Carlos III, Madrid, Spain; Medical Faculty, Otto-von-Guericke University Magdeburg, Medical Faculty, GERMANY

## Abstract

**Background:**

Meteorin (METRN) is a recently described neutrophic factor with angiogenic properties. This is a nested case-control study in a longitudinal cohort study that describes the serum profile of METRN during different periods of gestation in healthy and preeclamptic pregnant women. Moreover, we explore the possible application of METRN as a biomarker.

**Methods and Findings:**

Serum METRN was measured by ELISA in a longitudinal prospective cohort study in 37 healthy pregnant women, 16 mild preeclamptic women, and 20 healthy non-pregnant women during the menstrual cycle with the aim of assessing serum METRN levels and its correlations with other metabolic parameters. Immunostaining for METRN protein was performed in placenta. A multivariate logistic regression model was proposed and a classifier model was formulated for predicting preeclampsia in early and middle pregnancy. The performance in classification was evaluated using measures such as sensitivity, specificity, and the receiver operating characteristic (ROC) curve. In healthy pregnant women, serum METRN levels were significantly elevated in early pregnancy compared to middle and late pregnancy. METRN levels are significantly lower only in early pregnancy in preeclamptic women when compared to healthy pregnant women. Decision trees that did not include METRN levels in the first trimester had a reduced sensitivity of 56% in the detection of preeclamptic women, compared to a sensitivity of 69% when METRN was included.

**Conclusions:**

The joint measurements of circulating METRN levels in the first trimester and systolic blood pressure and weight in the second trimester significantly increase the probabilities of predicting preeclampsia.

## Introduction

Pregnancy is a period during which a woman experiences a series of anatomical, histological, hormonal, and metabolic changes that facilitate the development of a new human being [1]. As part of this process, it is necessary that a proper maternal-fetal interaction is developed so that the fetus can receive all the nutrients needed for growth [2]. Therefore, a balance between the angiogenic and anti-angiogenic processes in the placenta is critical because this new vascular network will be in charge of the fetal metabolic support, ensuring its viability [3].

During pregnancy, angiogenesis is an important and complex process involving several families of growth factors. Among these, two of the most documented are the family of vascular endothelial growth factor-A (VEGF-A) and the family of fibroblast growth factor-2 (FGF-2) [4,5]. On the other hand, meteorin (METRN) is a recently described neurotrophic factor that does not share homologous motifs with VEGF-A or FGF-2, thus forming a new family, named meterorin-like (METRNL), which conserves about 40% of its identity [6]. METRN is a secreted protein widely expressed in adult mouse organs [7,8]. It also promotes the formation of GFAP-positive glia via activation of the Jak-STAT3 pathway [9,10]. In addition to its role as a neurotrophic factor, METRN has been described as an important factor in attenuating angiogenesis at the gliovascular interface [8].

Preeclampsia is a leading and direct cause of maternal and infant morbidity and mortality worldwide. It consists of a multisystemic disorder between the maternal condition and the fetoplacental unit [11]. An imbalance in the ratio of angiogenic / anti-angiogenic factors is a well characterized feature of preeclampsia [11] and the implications that this imbalance may have in the occurrence of long-term complications in the offspring is not yet clear. Since METRN is a secreted protein that attenuates angiogenesis in astrocytes [8], we hypothesized that circulating METRN levels might be altered during normal pregnancy and preeclampsia. In the present work, using a longitudinal cohort study, we describe for the first time the serum METRN profile in pregnant women during different periods of normal pregnancy and in preeclamptic women. Thus, this study contributes to the understanding of the potential role of METRN in the physiology and pathology of pregnancy.

## Materials and Methods

### Subjects and Study design

The Ethics Committees of the Universidad Nacional de Colombia and the Hospital of Engativá approved this study. Written informed consent was provided by each of the women who participated in the study, with all the participants signing the informed consent to be included and could leave at any time. Also, the ethics committees followed this study and ensured participant safety during this investigation. This study was conducted by the Departments of Physiology and Obstetrics and Gynecology of the Faculty of Medicine of the Universidad Nacional de Colombia and Departments of Physiology of the University of Santiago de Compostela–Spain, and included pregnant patients of the Hospital of Engativá (Bogotá, Colombia).

We conducted a nested case-control study in the longitudinal cohort. Women were included in this study between the 11th and 13th week of gestation, determined by ultrasound and last menstrual period. This study included 37 healthy pregnant women, with no medical or obstetrical complications, delivering at term during the period of 2012–2014, whom were studied during early (11.6–12.6 weeks), middle (24.2–24.6 weeks) and late pregnancy (34.1–35.1 weeks) (Table 1).

**Table 1:** Clinical and biochemical characteristics of normal pregnant and preeclamptic women.

Variable	EP			MP			LP		
	Normal pregnant (n = 37)	PE pregnant (n = 16)	p-value	Normal pregnant (n = 37)	PE pregnant (n = 16)	p-value	Normal pregnant (n = 37)	PE pregnant (n = 16)	p-value
**Age. years (median(IQR))**	23 (20–31)	20.5 (19–26.7)	0.465						
**Weight. Kg. (mean +/-SD)**	55.6 (+/- 7.2)	60.8 (+/- 8.5)	0.041	60.1 (+/- 7.5)	66.2 (+/- 8.7)	0.021	65.1 (+/- 8.3)	73.7 (+/- 9)	0.003
**Body mass index. Kg/m2 (median(IQR))**	22.1 (20.7–24.3)	23.6 (21.6–25.5)	0.106	24.2 (22.5–26.2)	26 (23.7–28.1)	0.048	26.5 (+/- 2.6)	29.3 (+/- 3.2)	0.005
**Gestational age. weeks (median(IQR))**	12.3 (11.6–12.6)	12.3 (11.6–12.6)	0.976	24.3 (24.2–24.6)	24.1 (24–24.4)	0.277	34.4 (34.1–35.1)	34.9 (34.2–35.5)	0.118
**Gestational age at delivery. weeks (median(IQR)**							39 (38.4–39.6)	38(36.6–38.7)	0.000
**Systolic blood pressure. mmHg (mean+/SD)**	96.9 (+/- 8.7)	106.1 (+/- 6.3)	0.000	90(88–100)	100 (99–108.5)	0.012	93 (90–102)	109(100–120)	0.000
**Diastolic blood pressure. mmHg (median(IQR))**	60 (60–64)	65 (60–70)	0.109	60 (58–60)	61 (60–66.5)	0.055	62 (60–68.5)	65 (60–70)	0.412
**Mean arterial pressure. mmHg (mean+/SD)**	73.8 (+/- 6.1)	79.4 (+/- 6.3)	0.006	71.7 (+/- 6.8)	76.1 (+/- 4.9)	0.011	72.7 (70–78.3)	80(76.3–82.3)	0.009
**Glucose. mg/dL (mean+/-SD)**	77.5 (+/- 7.3)	80.5 (+/- 7.1)	0.171	74.6 (+/- 5.3)	76.6 (+/- 7.5)	0.348	73 (69–78)	73.5 (68.7–78.5)	0.538
**Insulin. uUI/mL (median(IQR))**	8.3 (5.2–11.7)	11.9 (10.5–13.7)	0.018	11.1 (+/- 4.6)	15.8 (+/- 6)	0.01	14.3 (+/- 5.3)	13.9(+/- 5.1)	0.807
**HOMA IR (median(IQR))**	1.7 (1–2.2)	2.4 (1.9–2.8)	0.008	1.9 (1.6–2.6)	2.8 (2.1–3.6)	0.013	2.6 (+/- 1.1)	2.6(+/- 1.1)	0.967
**Total cholesterol. mg/dL (mean+/-SD)**	168.5 (+/- 31.3)	171.4 (+/- 35)	0.774	217.6 (+/- 35.5)	218.2 (+/- 48.7)	0.964	241.2 (+/- 44)	228.2 (+/- 47.9)	0.357
**HDL-chol. mg/dL (mean+/-SD)**	56.8(+/- 10.8)	51.5(+/- 12.6)	0.160	70.6(+/- 12.2)	61.6(+/- 15)	0.045	68.1(+/- 12.5)	58.3 (+/- 17.2)	0.050
**Triglycerides. mg/dL (median(IQR))**	95.6(80.8–117.5)	109.8(77–154)	0.548	167.9 (132.8–201.7)	171.2 (133.2–219.5)	0.793	232.8(+/- 70.6)	240.5 (+/- 76)	0.731
**METRN. ng/mL (mean+/-SD)**	26.9(+/- 2.2)	24.2 (+/- 2.1)	0.000	24.9 (+/- 2.3)	25 (+/- 1.9)	0.939	25.2(+/- 2.2)	24.6(+/- 1.9)	0.330

EP: Early Pregnancy, MP: Middle Pregnancy, LP: Late Pregnancy, PE: Preeclamptic. Data with normal distribution were reported as mean +/- standard deviation (SD), while data with non-normal distribution were reported as median and interquartile range (IQR). A p-value < 0.05 was considered statistically significant.

Also, 16 women of the same longitudinal cohort who developed mild preeclampsia were included in this study (Table 1). The diagnosis of preeclampsia was defined as a persistent (> 6 h) high blood pressure > 140/90 mmHg, and proteinuria was defined as a urine protein concentration > 30 mg/dL in at least two specimens collected at least 4 hours apart, as described elsewhere [12]. Furthermore, the diagnosis of preeclampsia was confirmed by determining serum levels of the following factors: soluble endoglin (sEng) (ab100507), placental growth factor (PlGF) (ab100629), and soluble vascular endothelial growth factor receptor 1 (sVEGFR-1) (ab119613) [13]. Pregnant women with normal outcomes were used as a control (data not shown). The human ELISA kits were purchased from Abcam and the sample concentrations (ng/ml) utilized were in accordance with the manufacturer’s protocols.

Additionally, 20 young healthy non-pregnant women were studied during the follicular (3rd–5th day) and luteal phase (21st–23rd day) of the menstrual cycle (Table 2). The recruitment of healthy non-pregnant women occurred during the same period of recruitment as the other groups. The inclusion and exclusion criteria for participation in the study have been described elsewhere [12]. The demographic and clinical features of all the women are described in Table 1 and Table 2.

**Table 2:** Anthropometric and biochemical characteristics of healthy non—pregnant women.

Variable	non—pregnant women (n = 20)	p-value^a^
**Age, years (median (IQR))**	23 (20–26)	
**Body mass index, Kg/m2 (mean +/- SD)**	21.4 (+/- 2)	
**Insulin, uUI/mL (median (IQR))**	6 (4.4–12.5)	
**Glucose, mg/dL (mean +/- SD)**	82 (+/- 6.2)	
**HOMA IR (median (IQR))**	1.2 (0.8–2.7)	
**Total cholesterol, mg/dL (mean +/- SD)**	167.8 (+/- 22.9)	
**HDL-chol, mg/dL (mean +/- SD)**	47.3 (+/- 10.9)	
**Triglycerides, mg/dL (mean +/- SD)**	73.2 (+/- 19.2)	
**Progesterone, ng/mL (mean +/- SD) early follicular (cycle day 4 +/- 1)§**	0.6 (+/- 0.2)	<0.0001^b^
**Progesterone, ng/mL (mean +/- SD) median luteal (cycle day 22 +/- 1)§**	10.1 (+/- 5.9)	
**METRN, ng/mL (mean +/- SD) early follicular (cycle day 4 +/- 1)Ϯ**	23.3 (+/- 2.9)	0.2801^c^
**METRN, ng/mL (mean +/- SD) median luteal (cycle day 22 +/- 1)Ϯ**	24.1 (+/- 2.1)	

^a^ The difference between group means (early follicular and median luteal phases) was tested on progesterone and METRN levels. Only progesterone levels are significantly different between early follicular and median luteal phases (p<0.0001). Data with normal distribution were reported as mean +/- standard deviation (SD), while data with non-normal distribution were reported as median and interquartile range (IQR).

^b^ Progesterone determination in the two different phases of the menstrual cycle.

^c^ Meteorin measurement in luteal and follicular phase.

### Laboratory assays

Whole venous blood was drawn from the upper arm into BD Vacutainer serum tubes. Samples were centrifuged at 3000g for 10 minutes at 4°C and serum was aspirated out, aliquoted, and stored at -80°C until biochemical and hormonal assays were performed. Total cholesterol, HDL cholesterol, triglycerides, glucose, and insulin were measured (LIAISON Analyzer, Saluggia, Italy). Homeostasis Model Assessment–Estimated Insulin Resistance (HOMA-IR) was calculated according to Matthews’ *et al* formula [12]. Additionally, serum progesterone levels in healthy non—pregnant women during the luteal and follicular phases of the menstrual cycle were measured (Roche Elecsys 1010 Immunoanalyzer, Boulder,Colorado, USA).

The measurement of serum METRN concentrations was determined using a commercially available ELISA (Uscn Life Science Inc. Cat # SEH662Hu). The detection range of METRN was 0.156–10 ng/mL. Inter and intra-assay variation coefficient was 9% and 10% respectively. Additionally, the METRN concentration of each sample was analyzed in duplicate and the mean of the two measurements for each sample were reported in the statistical analysis.

### METRN immunohistochemistry in human placenta

Immunostaining for METRN was performed in embedded human adipose tissue and placenta. These paraffin blocks were provided from the Services of the Department of Pathology, Faculty of Medicine, at the Universidad Nacional de Colombia. Placental samples from patients at week 11 of gestation (from spontaneous abortion without histopathological alterations) were analyzed as described elsewhere [14].

Polyclonal rabbit anti–METRN antibodies (Abcam–Anti-METRN antibody–ab131619) were employed for the immunostaining, using a method described elsewhere [12]. With respect to the negative control a non-specific rabbit IgG was used in place of the primary polyclonal rabbit anti-METRN antibody in each immunohistochemistry placental specimen.

### Choice of criteria for predictive algorithm for preeclampsia assessment

In general terms, the diagnosis of preeclampsia was based on the criteria set forth by the American College of Obstetrics and Gynecology (ACOG). Based on the recommendations of the Task Force on Hypertension in Pregnancy by the American College of Obstetricians and Gynecologists, and due to the nature of preeclampsia, this expert committee eliminates the dependence on the presence or absence of proteinuria for the diagnosis of preeclampsia [15]. Therefore, in the absence of proteinuria preeclampsia is diagnosed in association with other clinical features of the syndrome such as: thrombocytopenia (a platelet count below 100,000 / uL), impaired liver function (altered levels of liver transaminase, doubling its normal value), development of renal failure (elevated levels of serum creatinine greater than 1.1mg / dL or a doubling of serum creatinine in the absence of other renal disease), pulmonary edema, or cerebral or visual disturbances [15]. This recommendation is due largely to a percentage of cases diagnosed with preeclampsia, in which proteinuria is undetected or of late onset [16]. In addition to the clinical monitoring of the patients in this cohort study, the characterization of this subpopulation was based on the analysis of serum pro and anti-angiogenic factors previously documented as early predictors of this disease. Based on previously described soluble endoglin (sEng), placental growth factor (PlGF) and soluble vascular endothelial growth factor receptor 1 (sVEGFR-1), on the serum profile both for normal pregnant and pregnant women diagnosed with preeclampsia was determined, and was found to be in agreement with what has already been reported by Romero *et al*. [13].

### Statistical Analysis

The data were analyzed using R software (version 3.0.3). The difference between group means (preeclampsia and normal) was tested on the demographic and clinical features at each pregnancy trimester. The Student's t-test was used when the variables were normally distributed; otherwise, the non-parametric Wilcoxon-Mann-Whitney test was used. Some variables were transformed to the natural logarithm to assure normal distribution. The normal distribution of data was verified using the Shapiro-Wilk test. A p-value < 0.05 was considered statistically significant. Data with normal distribution were reported as mean +/- standard deviation (SD), while data with non-normal distribution were reported as median and interquartile range (IQR).

A univariate analysis was performed to examine the correlation between serum METRN levels and the variables throughout pregnancy. Univariate correlations were assessed on the normal group by partial Spearman’s correlation coefficient with adjustment for gestational age.

With the aim of identifying independent relationships between the variables and preeclampsia, a multivariate logistic regression model was proposed. The logit model was selected to explain the odds of suffering preeclampsia as a function of the anthropometric and metabolic variables in the study. The Akaike information criterion (AIC) was used to select the parameters that best explain the variability in the dependent variable. Afterwards, the 95% confidence intervals for the odds of preeclampsia were assessed on significant parameters.

As a final step in our methodology, a classifier model was formulated for predicting preeclampsia in early and middle gestational ages, i.e first and second trimesters. The classification between preeclamptic and normal pregnant women was based on a decision tree approach. The decision tree was constructed in Weka (version 3.6.10) [17] using the J48 algorithm [18] with a 10-fold cross validation for testing predictions. Those variables showing significant differences between group means were included in the model. Moreover, their variations (delta variables) between the first and second trimesters were included. The performance in classification was evaluated using measures such as sensitivity, specificity, and the receiver operating characteristic (ROC) curve [19].

## Results

### Immunohistochemistry of METRN protein in human placenta

**Fig 1:**
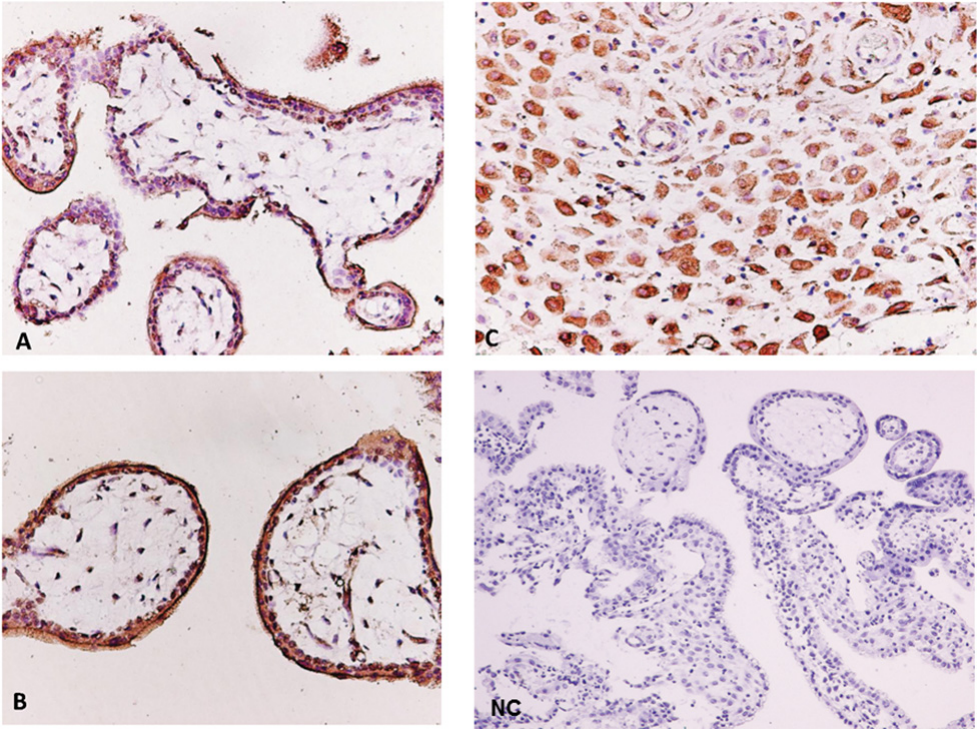
METRN immunohistochemisty in human placenta. Human placenta in the first trimester of pregnancy (spontaneous abortion) in which immature mesenchymal villi of first trimester decidua are illustrated. Moderate cytoplasmic immunoreactivity for METRN was observed in cytotrophoblast cells, syncytiotrophoblast cells and decidual cells (20x). A non-specific rabbit IgG was used as negative control in place of the primary polyclonal rabbit anti-METRN antibody in each immunohistochemistry placental specimen.

METRN immunostaining was observed in immature mesenchymal decidua and villi from first trimester human placenta (Fig 1A, 1B and 1C, respectively). Moderate cytoplasmic immunoreactivity was observed in cytotrophoblast, syncytiotrophoblast, and decidual cells. A non-specific rabbit IgG was used as a negative control in place of the primary polyclonal rabbit anti-METRN antibody in each immunohistochemistry placental specimen (Fig 1).

### Serum METRN levels in healthy non-pregnant women, normal pregnant and preeclamptic women

Measurement of clinical and biochemical characteristics of healthy and preeclamptic pregnant women and healthy non—pregnant women are shown in Tables 1 and 2. In addition, the characterization of preeclamptic subpopulation was based on the analysis of serum pro and anti-angiogenic factors, soluble endoglin (sEng), placental growth factor (PlGF) and soluble vascular endothelial growth factor receptor 1 (sVEGFR-1), and was found to be in agreement with what has already been reported by Romero *et al*. (data not shown) [13].

**Fig 2:**
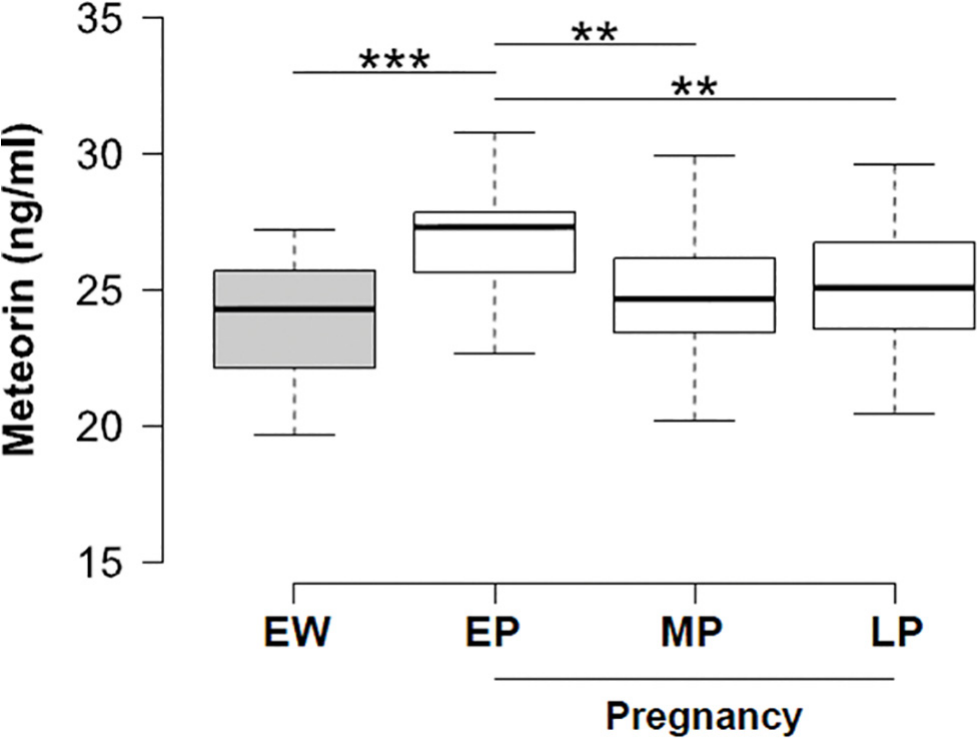
Serum METRN levels in the three trimesters of pregnancy and in a group of eumenorrheic woman. The highest levels of METRN are observed in early pregnancy (EP) and then decline with advancing gestation in the subsequent periods, in middle pregnancy (MP), and late pregnancy (LP), with this reduction in serum METRN being statistically significant (p <0.01). Moreover, significant differences were observed when comparing serum METRN in the groups of eumenorrheic women (EW) and EP (p <0.001).

Serum METRN levels were significantly higher in the first stage of gestation when compared to healthy non—pregnant women (Fig 2 and Fig C in S1 Fig). Circulating METRN levels were also significantly higher in the first trimester in comparison to the second and third trimesters of pregnancy (p <0.01) (Fig 2 and Table 1). Additionally, METRN levels in middle and late pregnancy were similar to the ones obtained in non-pregnant women (Fig 2). No statistically significant associations were observed between METRN concentrations in early pregnancy (EP) compared to middle (*r* = -0.043, *P* = 0.804) (MP) and late pregnancy (*r* = -0.266, *P* = 0.122) (LP) (Figs A and B in S1 Fig, respectively). In these figures, most of the patients are under the solid line (y = x), endorsing the conclusion that MERTN levels tend to be higher in EP than in MP or LP. Additionally, a dot plot for EP serum METRN concentration in normal pregnancy and eumenorrheic women (EW) is shown in Fig C in S1 Fig. The bar means values are also shown. As stated before, there is a significant difference between the groups (P<0.001).

**Fig 3:**
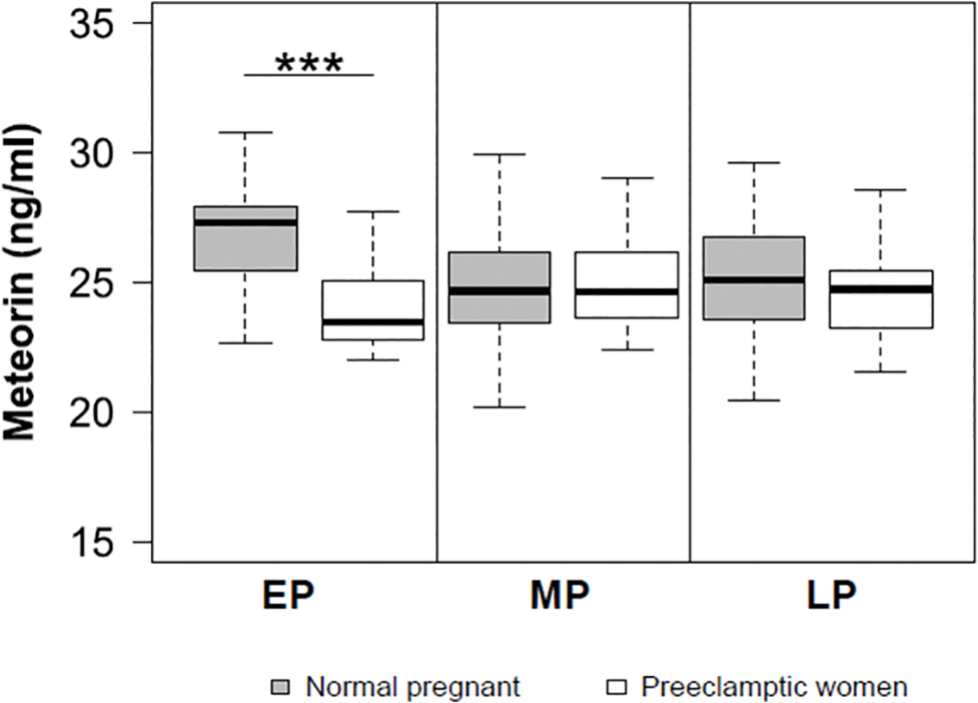
Serum METRN levels in healthy and preeclamptic women during pregnancy. A significant decrease in serum METRN in the group of mild preeclamptic pregnant women is observed when compared with the group of healthy pregnant women at the onset of pregnancy (EP) (p <0.001). The other periods analyzed did not show significant variations in serum METRN levels.

Furthermore, serum METRN levels were significantly higher in normal pregnant women in comparison to the preeclamptic pregnant women during the early period of gestation (p <0.0001) (Fig 3 and Table 1). However, no significant differences were found in serum METRN levels between normal pregnant women and preeclamptic pregnant women in the second and third trimesters (p >0.05) (Fig 3).

### Correlations between serum METRN levels and clinical and biochemical features

In normal pregnant women, univariate partial correlations were performed between serum METRN levels and the different clinical/biochemical parameters observed (S1 Table). Serum METRN levels were adjusted for gestational age in the univariate correlation analysis. Partial correlation analysis after adjustment showed that serum METRN levels concentrations were negatively associated with glucose levels (r = -0.39, p = 0.01) and positively associated with triglyceride levels (r = 0.34, p = 0.03) only in the first period of gestation. In contrast, serum METRN levels were not significantly correlated with weight, BMI, systolic blood pressure (SBP), diastolic blood pressure (DBP), mean arterial pressure, insulin, HOMA-IR, total cholesterol, HDL-cholesterol, and LDL cholesterol in any of the periods that were studied (S1 Table).

### Logistic regression analysis with preeclamptic condition as dependent variable

A logistic regression model was formulated and the parameters were summarized in S2 Table. The logit model links the binary response preeclampsia or not preeclampsia as a function of the predictors: gestational age (early, middle, or late pregnancy), BMI, SBP, triglycerides, and METRN levels. All these variables were found to be significant in explaining the odds of preeclampsia and a 95% confidence interval was assessed. In this logistic regression analysis, it should be noted that the odds of preeclampsia decrease between 1% and 35% when the METRN level increases 1ng/mL (S2 Table).

### Predictive algorithm for preeclampsia assessment

**Fig 4:**
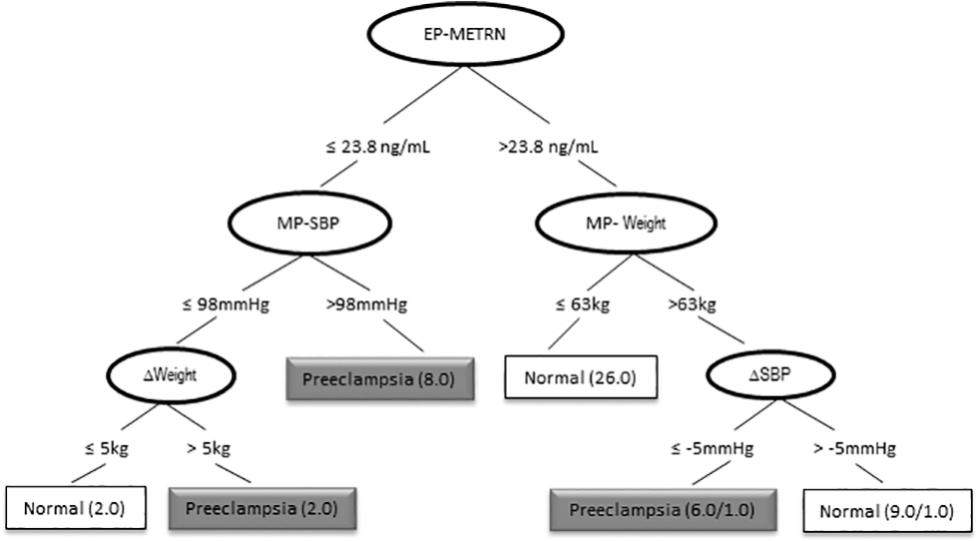
Classifier model based on decision trees. Decision nodes are represented by circles. Decision nodes show the significant variables used to perform the classification: EP-METRN (METRN levels in early pregnancy), MP-SBP (systolic blood pressure in middle pregnancy), MP-Weight (weight in middle pregnancy), ∆Weight (delta of weight between middle and early pregnancy: ∆Weight = MP.weight–EP.weight) and ∆SBP (Delta of Systolic Blood Pressure between middle and early pregnancy: ∆SBP = MP.SBP–EP.SBP). Each branch represents a test given to the decision node. Leaf nodes are represented by squares. Leaf nodes show the class in which a patient is classified (normal or preeclampsia). Leaf nodes also show the total of women from the cohort that were properly classified / total of women from the cohort misclassified.

A tree based approach was implemented to diagnose preeclampsia before the third period of pregnancy. We selected variables that showed significant differences (Table 1) in early pregnancy (weight, SBP, insulin, METRN, and HOMA) and middle pregnancy (weight, SBP, insulin, METRN, HOMA-IR, and HDL-cholesterol). In addition, the delta variables (weight, SBP, insulin, and HOMA-IR) were included (see statistical analysis). After applying the J48 algorithm, only noteworthy variables were included in the decision tree (Fig 4). The decision tree performs worthwhile classifications in both normal and preeclamptic conditions (S3 Table). After testing the decision tree with cross-validation, it shows a sensitivity of 69% and specificity of 76% in detecting preeclampsia. Moreover, the area under the ROC curve is 0.73, confirming a good level of accuracy in the classification (S2 Fig).

Serum METRN levels in early pregnancy is the first decision node (top of the tree) and therefore, the most important variable to classify. The classifier model proposes a critical METRN level of 23.8ng/mL. In the longitudinal cohort, ten out of sixteen women under the critical METRN levels developed preeclampsia (<23.8ng/mL). These ten women showed either high SBP in middle pregnancy (>98mm Hg) or a high increase of weight (**∆**weight > 5kg). The decision tree shows that all ten women under the critical METRN level were properly classified.

On the other hand, we found six preeclamptic women whose METRN levels exceeded the threshold (>23.8 ng/mL) in early pregnancy. These women showed not only increased weight in middle pregnancy (>63kg) but also their **∆**SBP decreased considerably (**∆**SBP < -5mmHg). In the decision tree, the classification over the right path is slightly less accurate (Fig 4). Five preeclamptic women from the cohort were properly classified and one was misclassified.

## Discussion

The results of this study reveal for the first time that serum METRN levels did not differ significantly in non-pregnant women studied during the follicular and luteal phase of the menstrual cycle. However, serum METRN levels are significantly elevated in the first trimester in healthy pregnant women whereas they decrease in the second and third trimesters reaching similar levels to the ones observed in non-pregnant women. Conversely, serum levels of METRN remained unchanged throughout gestation in preeclamptic women, being significantly lower in the first period of gestation when compared with serum levels of healthy pregnant women. Finally, we detected METRN protein immunoreactivity in the cytoplasm of cytotrophoblast cells, syncytiotrophoblast cells, and decidual cells in first trimester placenta.

Recent studies have shown that METRN participates in cerebral angiogenesis [8] as well as neurogenesis [4]. Previous studies in primary astrocytes identified expressed sequence tags that were increased under re-oxygenation following hypoxia [20,21]. This hypoxia/reoxygenation regulatory factor was identified as METRN. A previous study found that METRN blocks the angiogenic activity of microvascular endothelial cells, by induction of thrombospondin-1/-2 expression in astrocytes [8]. Elevated serum METRN levels in the first period of gestation might contribute to the adaptation of the fetal placental vasculature to the increasing fetal demands, enhancing the efficiency and high capacity of the maternal-fetal exchange system for the growth and development of the fetus.

Although METRN is widely expressed in rodent tissues, most studies have specifically focused on the functions of METRN in the central nervous system [6,9,22]. This is the first study demonstrating protein expression levels of this gene in human placenta, suggesting that METRN might play a critical role in controlling vascular angiogenesis and maturation in the placenta. In addition, the present study shows a reduction in maternal serum METRN levels during the initial phase of gestation in preeclamptic women in comparison to healthy pregnant women. Thus, it is plausible to hypothesize that reduced METRN levels contribute to the risk of preeclampsia characterized by reduced placental vascular development, leading to poor placental function and fetal growth compromise [11,23].

Moreover, it is well documented the utility of pro and anti-angiogenic factors as serum biomarkers in the early prediction of maternal and perinatal outcome. This is because one mechanism related to the development of preeclampsia is the alteration in placental perfusion, which leads to ischemia, a release of pro-inflammatory factors leading to platelet activation and endothelial dysfunction, producing the underlying clinical characteristics of the disease. Thus, a dysregulation of pro and anti angiogenic factors has been described which contributes significantly to the pathogenesis of preeclampsia, among these VEGF, sFlt-1, PlGF, endothelin, and endoglin [24]. Therefore, systolic blood pressure, anthropometric variables, and serum meteorin levels were established as significant in formulating the algorithm for the early prediction of preeclampsia

In support of this hypothesis, and by means of a logistic regression analysis, the present study shows that the odds of preeclampsia decrease between 1% and 35% when METRN levels increase by 1ng/ml. Additionally, the classification tree (Fig 4) showed that serum METRN levels above / below 23.8ng/mL are crucial in the early detection of developing preeclampsia, along with cutoff values for SBP (> 98mmHg) and weight (≤ 63kg) during middle pregnancy. In the present study, in 50% of cases of preeclampsia in the analyzed longitudinal cohort, METRN levels were lower than the critical value and were associated with high SBP in middle pregnancy (> 98mmHg).

The decision tree showed that the variables SBP and weight, analyzed individually, are not sufficient to classify a woman in the cohort as preeclamptic or normal. The inclusion of serum METRN levels during early pregnancy significantly increases the sensitivity for classification. Decision trees that did not include METRN had a reduced sensitivity of 56% in the detection of preeclamptic women, compared to a sensitivity of 69% in the actual decision tree (S3 Table and Fig 4). These observations point to a possible use of METRN as a potential biomarker for the development of preeclampsia. Nevertheless further studies with different cohorts are needed in order to corroborate these findings and to establish this parameter in the routine clinical setting. In addition, our data also indicates the need of discovering new biomarkers that, added to the decision tree assessed here, could increase the sensitivity of detection to levels that could make the early detection the development of preeclampsia with total accuracy feasible in the clinical setting.

## Conclusion

In conclusion, this study demonstrates that: a) serum METRN levels are unaltered throughout the menstrual cycle in healthy non-pregnant women; b) first trimester human placenta shows a specific immunostaining of METRN in the cytoplasm of cytotrophoblast, syncytotrophoblast, and decidual cells; c) serum METRN levels are increased in the first trimester in healthy pregnant women and remain unchanged throughout gestation in preeclamptic women; d) the measurement of circulating METRN in the first trimester together with systolic blood pressure and weight in the second trimester significantly increases the probabilities of predicting preeclampsia.

## Supporting Information

S1 Fig Scatter plot of serum METRN concentrations. Serum METRN concentrations (ng/ml) in early pregnancy (EP) compared to serum METRN concentrations (ng/ml) during **(Figure A)** middle pregnancy (MP) (*r* = -0.043, *P* = 0.804) and **(Figure B)** late pregnancy (LP) (*r* = -0.266, *P* = 0.122). The solid lines represent equal values of METRN in both stages (y = x). The dashed lines depict the linear regression between variables. **(Figure C)** Dot plot for EP serum METRN concentration in normal pregnancy and eumenorrheic women (EW). Each point represents a patient, and the bar mean values are also shown (P<0.001).
(TIFF)

S2 Fig Receiver operating characteristic curve (ROC) of the decision tree constructed in Weka (version 3.6.10) using the J48 algorithm. The ROC curve shows the sensitivity and (1-specificity) to detect preeclampsia. A 10-fold cross validation was used for testing predictions and obtaining the points on the ROC curve.
(TIFF)

S1 Table Univariate partial correlations between METRN and clinical/biochemical parameters. (DOCX)

S2 Table Logistic regression analysis with preeclamptic condition as dependent variable (DOCX)

S3 Table Accuracy of the decision tree by class (DOC)
