# Supplementary figures and images for: Maternal Serum Meteorin Levels and the Risk of Preeclampsia
Source: PLoS One. 2015 Jun 29;10(6):e0131013. doi: 10.1371/journal.pone.0131013 (PMC4487999; doi:10.1371/journal.pone.0131013)

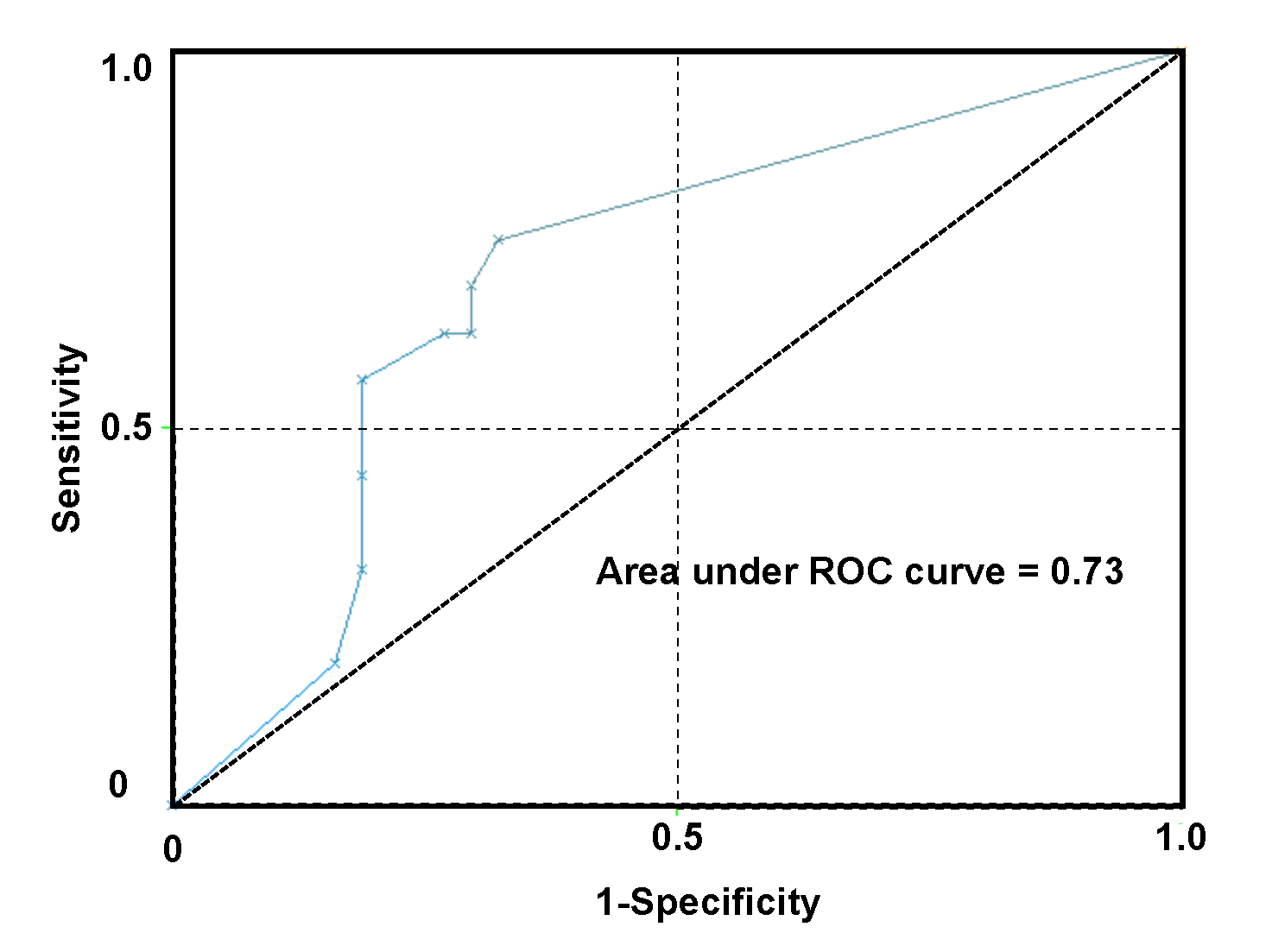

Supplement: S2 Fig — The ROC curve shows the sensitivity and (1-specificity) to detect preeclampsia. A 10-fold cross validation was used for testing predictions and obtaining the points on the ROC curve. (TIFF) [file pone.0131013.s002.tiff]
