# Supplementary material for: Maternal Serum Meteorin Levels and the Risk of Preeclampsia
Source: PLoS One. 2015 Jun 29;10(6):e0131013. doi: 10.1371/journal.pone.0131013 (PMC4487999; doi:10.1371/journal.pone.0131013)
Supplement: S1 Table — (DOCX) [file pone.0131013.s003.docx]

**S1 Table.** Univariate partial correlations between METRN and clinical/biochemical parameters**.**

| **Variable** | **EP** | | **MP** | | **LP** | |
| --- | --- | --- | --- | --- | --- | --- |
|  | **Spearman** | **p-value** | **Spearman** | **p-value** | **Spearman** | **p-value** |
| Weight | 0.01 | 0.94 | 0.03 | 0.87 | 0.04 | 0.82 |
| Height | 0.23 | 0.16 | 0.04 | 0.82 | 0.01 | 0.93 |
| BMI | -0.12 | 0.50 | -0.01 | 0.94 | -0.05 | 0.78 |
| Systolic blood pressure | 0.11 | 0.53 | -0.06 | 0.74 | -0.11 | 0.51 |
| Diastolic blood pressure | 0.12 | 0.50 | -0.14 | 0.42 | 0.02 | 0.92 |
| Mean arterial pressure | 0.12 | 0.47 | -0.15 | 0.37 | 0.02 | 0.92 |
| Glucose | -0.39 | **0.01^a^** | 0.09 | 0.60 | 0.02 | 0.90 |
| Insulin | 0.04 | 0.79 | -0.08 | 0.62 | 0.23 | 0.18 |
| HOMA IR | -0.03 | 0.88 | -0.06 | 0.72 | 0.20 | 0.24 |
| Total cholesterol | 0.20 | 0.23 | 0.06 | 0.74 | -0.08 | 0.63 |
| HDL-chol | 0.04 | 0.82 | 0.24 | 0.16 | -0.13 | 0.45 |
| LDL-chol | 0.26 | 0.12 | 0.18 | 0.27 | -0.09 | 0.62 |
| Triglycerides | 0.34 | **0.03^a^** | -0.13 | 0.45 | 0.31 | 0.06 |

EP: Early Pregnancy, MP: Middle Pregnancy, LP: Late Pregnancy. A p-value < 0.05 was considered statistically significant.
