# Supplementary material for: Maternal Serum Meteorin Levels and the Risk of Preeclampsia
Source: PLoS One. 2015 Jun 29;10(6):e0131013. doi: 10.1371/journal.pone.0131013 (PMC4487999; doi:10.1371/journal.pone.0131013)
Supplement: S2 Table — (DOCX) [file pone.0131013.s004.docx]

**S2 Table.** Logistic regression analysis with preeclamptic condition as dependent variable

| **Variable** | **Estimate** | **Standard Error** | **p-value** | **Significance**  **Code** | **O.R. Confidence interval (95%)** | |
| --- | --- | --- | --- | --- | --- | --- |
|  |  |  |  |  | **Lower Limit** | **Upper limit** |
| Model intercept | -20.13 | 5.26 | 0.0001 | *** | - | - |
| Early pregnancy | 2.54 | 0.87 | 0.0036 | ** | 2.30 | 70.42 |
| Middle pregnancy | 1.38 | 0.66 | 0.0350 | * | 1.10 | 14.42 |
| BMI^a^ | 0.23 | 0.08 | 0.0052 | ** | 1.07 | 1.48 |
| SBP^b^ | 0.16 | 0.03 | <0.0000 | *** | 1.10 | 1.25 |
| Triglycerides | 0.01 | 0.00 | 0.0059 | ** | 1.00 | 1.02 |
| METRN^c^ | -0.22 | 0.11 | 0.0371 | * | 0.65 | 0.99 |

**^a^** Body mass index **^b^**Systolic blood pressure ^c^ METRN. A p-value < 0.05 was considered statistically significant.
