# Supplementary material for: Maternal Serum Meteorin Levels and the Risk of Preeclampsia
Source: PLoS One. 2015 Jun 29;10(6):e0131013. doi: 10.1371/journal.pone.0131013 (PMC4487999; doi:10.1371/journal.pone.0131013)
Supplement: S3 Table — (DOC) [file pone.0131013.s005.doc]

**S3 Table.** Accuracy of the decision tree by class

| **Class** | **True Positive Rate** | **False Positive Rate** | **ROC Area** |
| --- | --- | --- | --- |
| Normal | 0.76 | 0.31 | 0.73 |
| Preeclampsia | 0.69 | 0.24 | 0.73 |
